# Structural and Biochemical Studies of a Moderately Thermophilic Exonuclease I from *Methylocaldum szegediense*

**DOI:** 10.1371/journal.pone.0117470

**Published:** 2015-02-06

**Authors:** Li Fei, SiSi Tian, Ruth Moysey, Mihaela Misca, John J. Barker, Myron A. Smith, Paul A. McEwan, Ewa S. Pilka, Lauren Crawley, Tom Evans, Dapeng Sun

**Affiliations:** 1 New England Biolabs Shanghai R&D Center, Building 5, 917 Halei Road, Pudong District, Shanghai, China; 2 Oxford Nanopore Technologies Ltd., Edmund Cartwright House, 4 Robert Robinson Avenue Oxford Science Park, Oxford OX4, United Kingdom; 3 Evotec (UK) Ltd, 114 Innovation Drive, Milton Park, Abingdon, Oxfordshire OX14 4RZ, United Kingdom; 4 New England Biolabs, 240 County Road, Ipswich, MA 01938–2723, United States of America; Institute of Enzymology of the Hungarian Academy of Science, HUNGARY

## Abstract

A novel exonuclease, designated as MszExo I, was cloned from *Methylocaldum szegediense*, a moderately thermophilic methanotroph. It specifically digests single-stranded DNA in the 3ʹ to 5ʹ direction. The protein is composed of 479 amino acids, and it shares 47% sequence identity with *E. coli* Exo I. The crystal structure of MszExo I was determined to a resolution of 2.2 Å and it aligns well with that of *E. coli* Exo I. Comparative studies revealed that MszExo I and *E. coli* Exo I have similar metal ion binding affinity and similar activity at mesophilic temperatures (25–47°C). However, the optimum working temperature of MszExo I is 10°C higher, and the melting temperature is more than 4°C higher as evaluated by both thermal inactivation assays and DSC measurements. More importantly, two thermal transitions during unfolding of MszExo I were monitored by DSC while only one transition was found in *E. coli* Exo I. Further analyses showed that magnesium ions not only confer structural stability, but also affect the unfolding of MszExo I. MszExo I is the first reported enzyme in the DNA repair systems of moderately thermophilic bacteria, which are predicted to have more efficient DNA repair systems than mesophilic ones.

## Introduction

Exonuclease I (Exo I) from *E. coli* is an Mg^2+^ ion dependent exonuclease, which specifically degrades single-stranded DNA (ssDNA) from 3' to 5' in a highly processive manner [1,2]. It is involved in several important DNA repair systems in *E. coli*, such as RecBCD-dependent homologous recombination, methyl-dependent mismatch repair, and genomic tandem repeat maintenance [3,4].

Extensive research has focused on elucidating the mechanism for its high processivity [5,6]. Recently, the crystal structure of Exo I complexed with ssDNA revealed the topological linkage and two-site substrate binding underlying the high processivity [6].

Several lines of evidence indicate that Exo I digests ssDNA by a two-metal ion catalyzed mechanism [5–8], which was previously described for the 3'-5' exonuclease domain of DNA polymerase I [9,10]. Both Exo I and the exonuclease domain of DNA polymerase I belong to the DnaQ superfamily (or DEDD superfamily) based on sequence analysis [5,11,12]. According to the two-metal ion mechanism, two magnesium ions are essential for exonuclease activity, with Mg^B^ binding directly to the enzyme and Mg^A^ binding via the 3' end of the bound ssDNA substrate [6,9]. The contribution of two metal ions to thermal stability of Exo I has not been characterized.

Currently, *E. coli* Exo I is a popular commercial product for the removal of PCR primers prior to DNA sequencing. The potential use of Exo I in nanopore DNA sequencing has also been explored owning to its high processivity and unusual resistance to high salt [13]. However, *E. coli* Exo I is a mesophilic enzyme with limited tolerance to higher temperature. To expand the use of Exo I in various applications, more robust enzymes are required. Exo I is conserved in bacteria but not in archaea, which makes it difficult to find a thermophilic ortholog. PfuExo I was recently found in the hyperthermophilic archaeon, *Pyrococcus furious*. Although it works well at high temperature, PfuExo I hydrolyzes ssDNA at every two nucleotides [14], adopting a different mechanism to *E. coli* Exo I.

Few investigations have focused on the metabolism of nucleic acids in moderately thermophilic bacteria. It is of great interest to know whether their way of metabolizing nucleic acids is similar to that of mesophilic bacteria or hyperthermophilic archaea. Here, a moderately thermophilic Exo I, which shares sequence similarity to *E. coli* Exo I, was found in *Methylocaldum szegediense* [15], whose growth temperature is 57°C. It has similar metal ion binding affinity, and similar activity at mesophilic temperatures as *E. coli* Exo I. The x-ray crystal structure of MszExo I was determined at a resolution of 2.2 Å and it was similar to that of *E. coli* Exo I. However, MszExo I has a broader working temperature and higher thermal stability. More importantly a magnesium ion was found to modulate its thermal unfolding curve as measured by differential scanning calorimetry (DSC).

## Materials and Methods

### DNA substrates

The following oligonucleotides were synthesized by Sangon Biotech (Shanghai, China): FRET-dT67, 67-nt poly dT labeled by a BHQ1 quenchor [16] on dT1 which is the first nucleotide from 5’ end and a fluorescein (6-FAM) donor on dT15 which is the fifteenth nucleotide from 5’ end; dT67, 67-nt poly dT; d22, 5'-AATTCGTGCAGGCATGGTAGCT-3'; d27, 5-AGCTATGACCATGATTACGAATTGCTT-3'; 5FAM-d49, 5’-6-FAM-AGCTACCATGCCTGCACGAATTAAGCAATTCGTAATCATGGTCATAGCT-3'; 3FAM-d49, 5'-AGCTACCATGCCTGCACGAATTAAGCAATTCGTAATCATGGTCATAGCT-6-FAM-3’; 5FAM-dA28, 28-nt poly dA labeled by 6-FAM at 5’ end; 5FAM-dC28, 28-nt poly dC labeled by 6-FAM at 5’ end; 5FAM-dT28, 28-nt poly dT labeled by 6-FAM at 5’ end. The 5’-overhang DNA (d27 and 3FAM-d49), and 3’-overhang DNA (d22 and 5FAM-d49) were generated by annealing the oligonucleotides in the annealing buffer containing 10 mM Tris-HCl, pH 7.5, 50 mM NaCl.

### Cloning MszExo I from *Methylocaldum szegediense*

The MszExo I gene (accession number: KM884769) was amplified from genomic DNA of *Methylocaldum szegediense* and 6*his-tag was attached to the C-terminus by PCR. The PCR amplifications were performed with Phusion DNA polymerase (New England Biolabs) for 28 cycles of 10 s at 98°C, 30 s at 60°C, and 30 s at 72°C. The final PCR product was digested with NdeI and SacI (New England Biolabs), ligated into placzz1 (a pUC19 derivative plasmid with a multiple-cloning site) vector, and transformed into *E. coli* strain BL21 (DE3). Positive clones were confirmed by DNA sequencing. The *E. coli* Exo I gene was amplified from the genomic DNA of *E. coli* strain BL21 (DE3) and ligated into pET21a overexpression vector (Invitrogen) with a 6*his-tag appended on the C-terminus of the protein.

### Protein expression and purification

The *E. coli* strains harboring the MszExo I (or *E. coli* Exo I) expression plasmid were grown at 37°C in LB medium containing 100 μg/ml ampicillin. When the OD600 value reached 0.6, the expression of recombinant protein was induced by 1 mM IPTG at 16°C for 16 hours. Cells were harvested and suspended in buffer A (50 mM Tris-HCl, pH7.5, 500 mM NaCl). Cells were disrupted by sonication on ice and the cell lysate was centrifuged (38,000 × g) for one hour at 4°C to remove insoluble materials. After centrifugation, the supernatant was collected, filtered by passing through a membrane filter (0.45 μm, Life Sciences) and loaded on a nickel column (Qiagen) equilibrated with buffer A. The protein was eluted with a linear gradient of 20–500 mM imidazole in buffer A. To get better purity, the resultant fractions containing target protein were collected and diluted 10-fold by buffer C (50 mM Tris-HCl, pH 7.5) before loading on a Q-sepharose anion exchange column (GE Healthcare Life Sciences) for *E. coli* Exo I or heparin agarose column (GE Healthcare Life Sciences) for MszExo I. The protein was eluted with a linear gradient of 50–1000 mM NaCl. Fractions containing the protein of interest were collected and dialyzed against buffer S (10 mM Tris-HCl, pH 7.5, 100 mM NaCl, 0.5 mM EDTA, 50% Glycerol) for storage. Protein concentrations were determined by measuring its absorbance at 280 nm using a calculated extinction coefficient of 60850 M^-1^cm^-1^for MszExo I and 74830 M^-1^cm^-1^ for *E. coli* Exo I.

### Exonuclease Assay

The exonuclease activity was measured by incubating 5 nM MszExo I with 50 nM various 6-FAM-labeled DNA substrates in the standard reaction buffer containing 50 mM Glycine-NaOH pH 9.5, 200 mM NaCl, 0.1 mg/ml BSA, 5 mM MgCl_2_ at 37°C. Reaction time is described in the figure legend. Reactions were quenched by 20 mM EDTA. The digestion products were visualized by Typhoon Trio variable mode imager (GE Healthcare Life Sciences) after electrophoretic separation on 20% (W/V) polyacrylamide-7 M urea gels.

### Endonuclease Assay

MszExo I (100 nM) was incubated with 25 ng/μl M13mp18 circular ssDNA (New England Biolabs) or Φ174 plasmid (New England Biolabs) in the standard reaction buffer for 1 hour at 37°C. The reaction was quenched by 20 mM EDTA. The products were separated on a 1% agarose gel, and visualized by GoldView staining.

### RNase Assay

The RNase activity was detected by using RNase contaminant kit ((New England Biolabs). MszExo I (100 nM) was incubated with 4 ng/μl 6-FAM-labeled RNA substrate from the kit in the standard reaction buffer for 1 hour at 37°C. The reaction was quenched by 20 mM EDTA. The products were visualized Typhoon Trio variable mode imager (GE Healthcare Life Sciences) after electrophoretic separation on 6% (W/V) polyacrylamide-7 M urea gels.

### Crystallization and structure determination

For crystallization studies the MszExo I gene was synthesized with an N-terminal cleavable strep tag. The preparation of MszExo I protein for crystallization is described in supporting information (S1 File). *Apo* MszExo I crystals were grown by mixing the protein at a concentration of 8 mg/ml with 20% PEG6K, 0.1 M Tris-HCl pH8, 0.2 M calcium chloride at a protein to precipitant ratio of 1:1. Crystals appeared within 3 days, and grew to a suitable size for data collection after 10–14 days. MszExo I crystals were cryo-protected by dipping them into a solution consisting of the components used for crystallization supplemented with 20% glycerol for 2–5 sec followed by quick flash freezing in liquid nitrogen and storage for data collection.

Diffraction data were collected at beamline I02 of Diamond Light Source, United Kingdom. Data was processed using the XIA2 pipeline. The *apo* MszExo I structure was solved by molecular replacement (using PHASER) with *E. coli* Exo I structure as the starting model (PDB ID: 1FXX). COOT visualization software was used for model building and REFMAC5 for refinement. Selected data collection and refinement statistics are given in Table 1.

**Table 1:** Data collection and refinement statistics.

Data collection	
Beamline	I02
wavelength (Å)	0.9795
space group	P2_1_2_1_2_1_
cell dimensions	
a, b, c (Å)	60.95 97 111.39
α, β, γ (^o^)	90 90 90
Resolution (Å)	2.183
Rmerge	4.30%
Mean I/σI	18.3
completeness (%)	99.22
Redundancy	4
Refinement	
Resolution (Å)	2.12
No.reflections	36277
*R*work/*R*free	22.2/25.3
No.atoms	4010
B-factors (Å^2^): protein	49.365
r.m.s. deviations	
Bond lengths (Å)	0.005
Bond angles (^o^)	1.029

### Fluorescence resonance energy transfer (FRET)-based exonuclease activity assay

The DNA substrate FRET-dT67 was dissolved in standard reaction buffer and diluted by dT67 at a ratio of 1:1. The FRET-based exonuclease activity was measured in a standard mixture containing 50 mM Glycine-NaOH pH 9.5, 200 mM NaCl, 0.1 mg/ml BSA, 5 mM MgCl_2_, 1 μM ssDNA (50% FRET-dT67 + 50% dT67), and 5–27 nM MszExo I or *E. coli* Exo I at 25 to 67°C. The reaction was triggered by the rapidly mixing of 90 μl of the enzyme in reaction buffer to 5 μl of substrate, which was preloaded in a 96-well plate. The degradation of ssDNA was instantly measured from continuous increase of fluorescence intensity at 522 nm excited by 492 nm for one minute on the Synergy Mx monochromator-based multi-mode microplate reader (Bioteck), controlled by Gene 5 software. The initial velocity of the reaction was obtained by calculating the slope of the initial linear portion of the curve.

### Determination of metal ion binding affinity (K_obs,Mg_) of *E. coli* Exo I and MszExo I by kinetic reaction

The initial velocities of exonuclease-catalyzed reactions were measured in the presence of 0 to 5 mM MgCl_2_. The temperature was controlled at 25°C by the microplate reader, and the protein was pre-incubated at 25°C for 30 sec on thermal cycler before assaying. The initial velocity data were fitted to the equation (2)$\;V_{o}\;=\;\frac{V_{max}*{[MgCl}_{2}]}{K_{obs,Mg}+\;{[MgCl}_{2}]}$ to obtain parameters K_obs,Mg_ and V_max_. In this equation, the initial velocity is represented as “V_o_”, the maximum of initial velocity represented as “V_max_”, and the observed dissociation constant for binding of two magnesium ions to the enzyme represented as K_obs,Mg_. Here, V_max_ is the initial velocity the enzyme would reach if both magnesium binding sites were fully saturated. The DNA substrate is already in a saturated condition.

### Effect of temperature on exonuclease activity

Activities of *E. coli* Exo I and MszExo I were determined over a temperature range of 25–67°C. Samples were pre-incubated at a given temperature (from 25 to 67°C) on the thermal cycler for 30 sec before the activity assay. The reaction was triggered by rapidly mixing of enzymes to ssDNA substrate (50% FRET-dT67 + 50% dT67) preloaded in a 96-well plate. The increase of fluorescence intensity at 522 nm was continuously monitored by the microplate reader controlled at the given temperature.

### Thermal inactivation assay

Enzymes were diluted in exonuclease reaction buffer to a final concentration of 15 nM. 100 μl of enzyme was incubated at fixed temperatures varying from 37 to 62°C for 10 min. Samples were immediately cooled on ice for 1 min, then incubated at 37°C for 30 sec followed by activity assay at this temperature. Data were normalized by taking the activity of Exo Is incubating at 37°C as 100%.

### Differential Scanning Calorimetry

DSC measurements were carried out on a MicroCal VP-capillary DSC differential scanning calorimeter (GE Healthcare Life Sciences) with an integrated autosampler. The DSC system was controlled by VPviewer program. Exo I samples (70 μM) were equilibrated in different buffers by dialysis before DSC measurement. Samples were analyzed by a programmed scan rate of 1.5°C/min over a temperature range of 10–110°C. Data were corrected by subtracting buffer baseline and normalized for protein concentration. T_m_ value was obtained by fitting *E. coli* Exo I data to a two-state model and MszExo I data to a non-two-state model or two-state model using the Microcal origin software. DSC data of MszExo I in buffers without magnesium ions were transformed into F_U_ (fraction unfolded)-T plots by integration [17].

## Results

### MszExo I is an ssDNA-specific 3’ to 5’ exonuclease

Sequence identities between MszExo I and *E. coli* Exo I are 47%, which suggests that MszExo I is an ssDNA-specific exonuclease (S1 Fig.). To test this prediction, nuclease assays were performed using 3’and 5’-overhang DNAs as substrates. Highly purified MszExo I (S2 Fig.) showed poor activity and thermal stability in the commercial buffer for *E. coli* Exo I (67 mM Glycine-KOH pH 9.5, 6.7 mM MgCl_2_, 10 mM β-mercaptoethanol, 0.1 mg/ml BSA), which contains barely no salt (data not shown). The performance of MszExo I increased significantly in the standard reaction buffer (50 mM Glycine-NaOH pH 9.5, 5 mM MgCl_2_, 200 mM NaCl, 0.1 mg/ml BSA) and further increase of sodium chloride made little difference.

**Fig 1:**
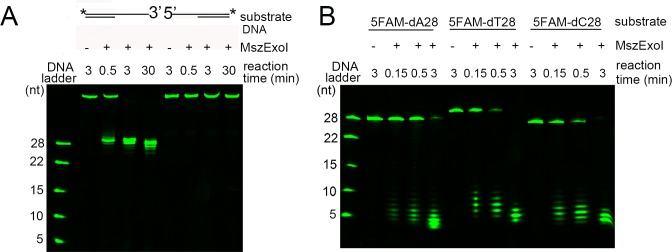
Substrate specificity of MszExo I. (A) MszExo I (5 nM) was incubated with 3’-overhang and 5’-overhang DNAs (50 nM) for indicated time at 37°C. The positions labeled by a 6-FAM are indicated by an asterisk for the DNA substrates. The 28-nt, 22-nt, 15-nt, 10-nt, and 5-nt poly dAs labeled by a 6-FAM at 5’-end were used as markers. Other details are described in Materials and Methods. (B) Time course experiments of the exonuclease activity of MszExo I were performed using 6-FAM labeled ssDNAs (5FAM-dA28, 5FAM-dT28 and 5FAM-dC28) as substrates. MszExo I (5 nM) was incubated with the DNA substrates (50 nM) at 37°C for indicated time. The 28-nt, 22-nt, 15-nt, 10-nt, and 5-nt poly dAs labeled by a 6-FAM at 5’-end were used as markers. Other details are described in Materials and Methods.

The degradation of substrates was only observed from the 3’-overhang DNA, and no cleavage was happened to the 5’-overhang DNA (Fig. 1A). This result supports the prediction that MszExo I is an ssDNA-specific exonuclease with 3’ to 5’ polarity. To further study substrate specificity of MszExo I, 5FAM-dA28, 5FAM-dT28 and 5FAM-dC28 were subjected to MszExo I treatment. The products were ladder-like bands at positions of lower than the 10-nt band for all three substrates (Fig. 1B). Probably, this was caused by the fact that the binding affinity of MszExo I to ssDNA decreased significantly when the length of ssDNA was shorter than 10 nucleotides (data not shown). These results indicate that the cleavage mode of MszExo I is processive. Interestingly, the ladder-like bands corresponded to every nucleotide judged from the position of the size marker bands, 10 nt and 5 nt. These results indicate the cleavage mode of MszExo I is similar to *E. coli* Exo I instead of PfuExo I.

**Fig 2:**
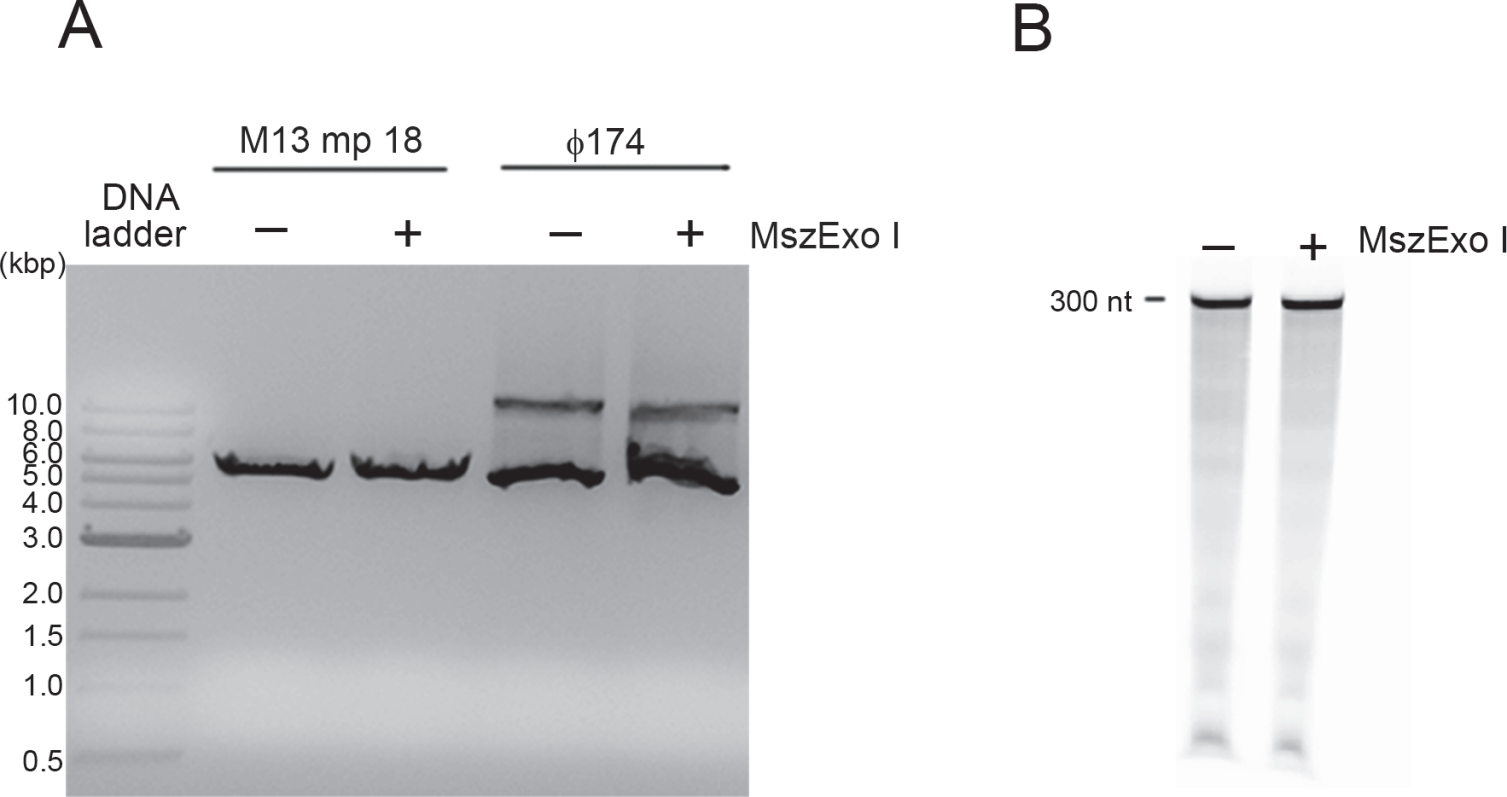
Endonuclease and RNase assay of MszExo I. (A) MszExo I (100 nM) was incubated with circular ssDNA (M13 mp18) or dsDNA (Φ174) in the standard reaction buffer at 37°C for 1 hour. The products were analyzed by electrophoresis on a 1% agarose gel which was then stained by GoldView. (B) In the RNase assay, the 300-nt RNA internally labeled by a 6-FAM was used as the substrate. The reaction was performed at 37°C for 1 hour in the standard reaction buffer. Samples were resolved on a 6% PAGE with 7 M urea. The products were visualized by Typhoon Trio variable mode imager.

To determine whether MszExo I has endonuclease activity, endonuclease assays were performed using M13 mp18 which is a circular ssDNA and Φ174 which is a circular double-stranded DNA (dsDNA) as substrates. No cleavage was observed with either circular ssDNA or dsDNA after incubation at 37°C for 1 hour (Fig. 2A). To detect whether MszExo I has RNase activity, the RNase assay was performed using the 300-nt RNA internally labeled by 6-FAM as the substrate. There was no digestion even after incubation at 37°C for 1 hour (Fig. 2B). These results indicate MszExo I has no endonuclease activity, nor RNase activity.

### X-Ray crystal structure of MszExo I

**Fig 3:**
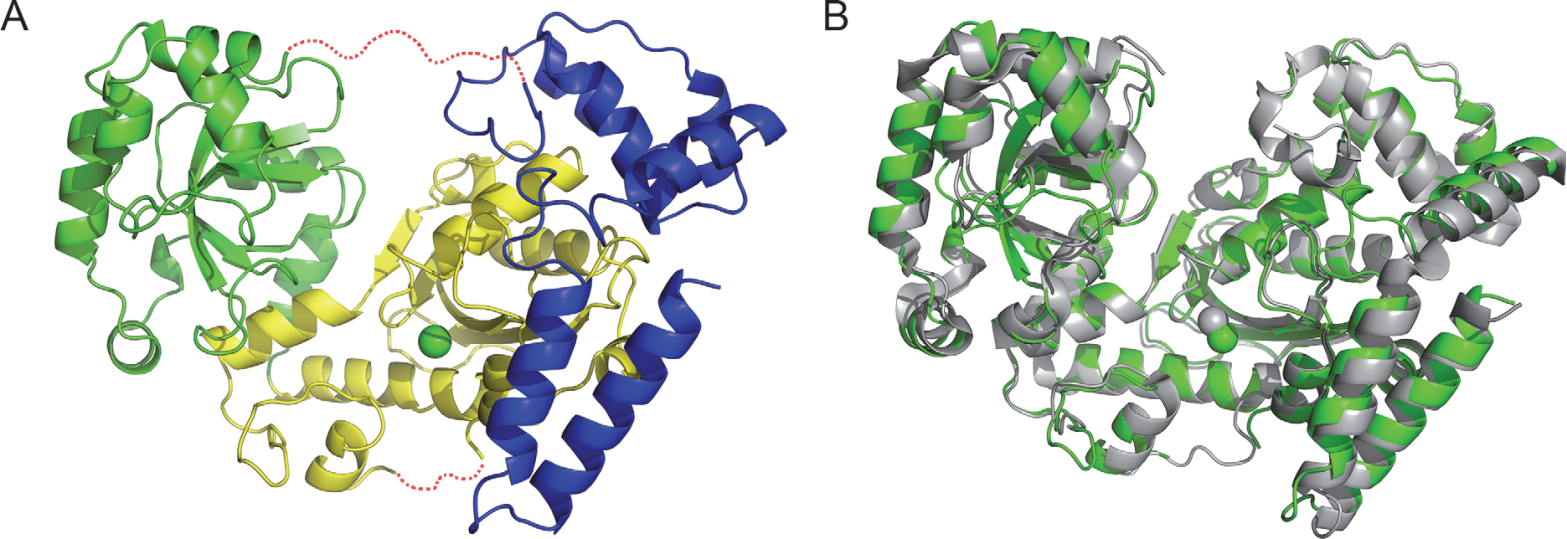
Structural alignment between *E. coli* Exo I and MszExo I. (A) Overall structure of MszExo I. Like *E. coli* Exo I, MszExo I can be divided into three domains, the N-terminal exonuclease domain (1–197) colored in yellow, the SH3-like domain (198–355) colored in green and the C-terminal helical region (356–479) colored in blue. The magnesium ion at the active site is represented as a green sphere. Two missing regions (173–177, 350–359) are represented as red dashed lines. (B) Structure alignment of *E. coli* Exo I and MszExo I. *E. coli* Exo I (PDB: 1FXX) is colored in grey, and MszExo I (PDB: 4RG8) colored in green. Images were generated using the Pymol program.

The results of activity assays and sequence alignment indicate MszExo I is an *E. coli* Exo I homolog in *Methylocaldum szegediense*. It would be interesting to know whether they have similar three-dimensional structures. The structure of MszExo I (PDB: 4RG8) was solved at 2.2 Å. The overall structure adopted a doughnut-like shape with a large groove through the center of the molecule, closely resembling *E. coli* Exo I (Fig. 3A). MszExo I is composed of three domains according to protein sequence alignment (Fig. 3A). The N-terminal exonuclease domain (residues 1–197) is located in the middle of the molecule. It is flanked by the SH3-like domain (residues 198–355) and C-terminal helical region (residues 356–479). A flexible region (residues 350–359) missing in the structure is predicted to behave as a linker to join the SH3-like domain and C-terminal domain (Fig. 3A). Residues at active site and anchor site, both of which are involved in substrate binding, are highly conserved among MszExo I and *E. coli* Exo I (S1 Fig). A single magnesium ion (Mg^B^) was observed at the active site, coordinated by Asp11 and Asp104. Glu13 and Asp182 are predicted to bind the other magnesium ion (Mg^A^), which was not present in the crystal structure probably due to the absence of substrate. Site-directed mutagenesis study showed that D104A and D182A mutants were devoid of exonuclease activity (data not shown), supporting the two-metal ion mechanism in 3' to 5' exonucleases.

Structure alignment of two Exo Is showed they were very similar in overall structure. The superposition of MszExo I and *E. coli* Exo I yields RMSD value for α-carbon atoms of 0.98 Å over 358 residues (Fig. 3B).

### Similar metal ion binding affinity is observed in MszExo I and *E. coli* Exo I

Mg^B^ was observed in the crystal structure of *apo* MszExo I. In *E. coli* Exo I, the corresponding magnesium ion is tightly bound, so that even incubation with 10 mM EDTA does not remove it entirely from the enzyme [6].

**Fig 4:**
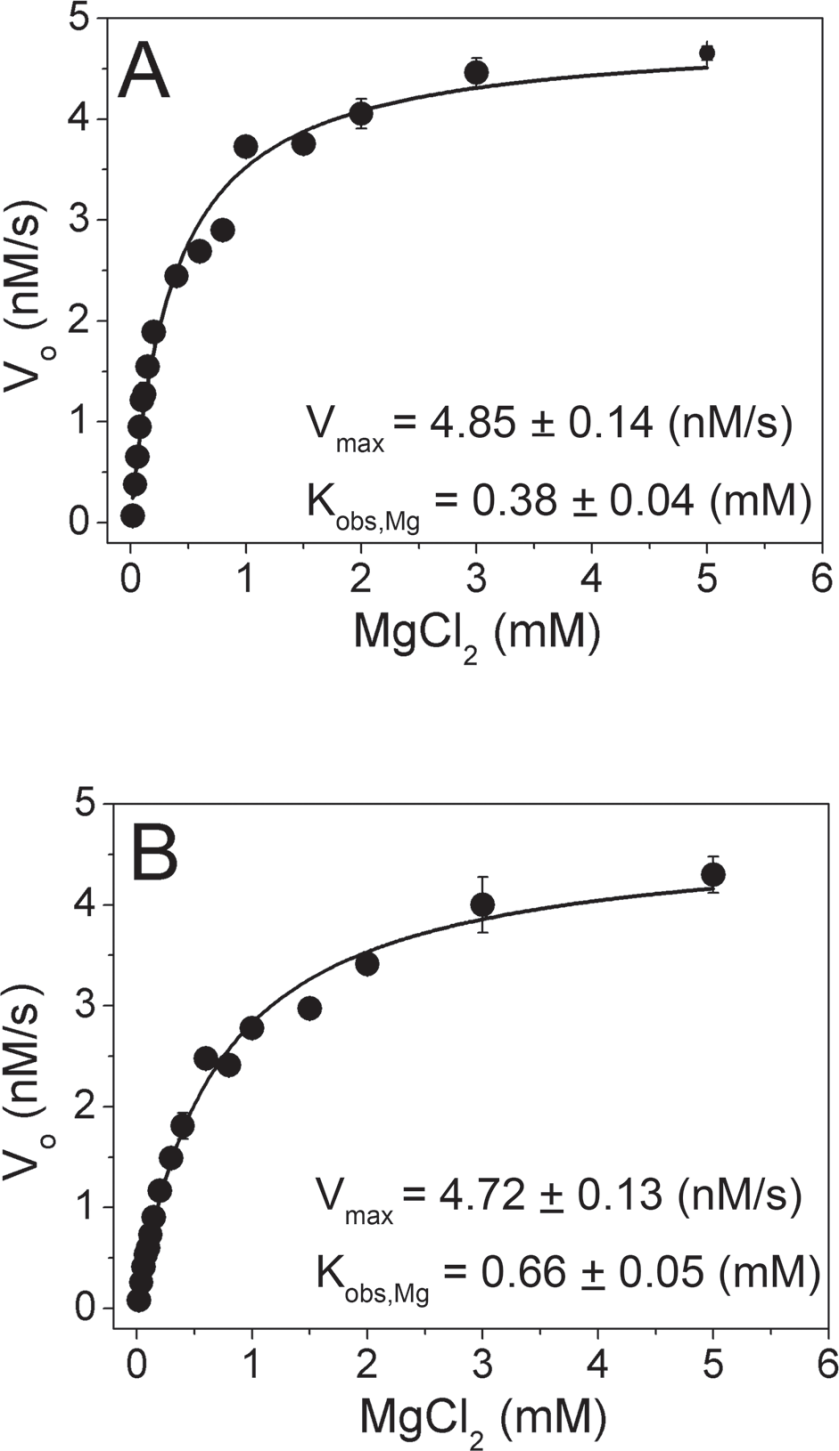
Determination of the metal ion binding affinity (K_obs,Mg_) of *E. coli* Exo I and MszExo I by kinetic reaction. The reaction conditions were 27 nM enzyme, 1 μM ssDNA substrate (half FRET-dT67 with half dT67), 50 mM Glycine-NaOH buffer, pH 9.5, 200 mM NaCl, 0.1 mg/ml BSA and 0–5 mM MgCl_2_ at 25°C. Initial velocity data were fitted to binding equation (2) as described in Materials and Methods, and then parameters V_max_ and K_obs,Mg_ were determined. Their values were indicated in the figure. (A) *E. coli* Exo I, (B) MszExo I. Errors were calculated from three parallel experiments.

Sequence alignment suggests that Glu13 and Asp182 in MszExo I bind Mg^A^, which is also essential for exonuclease activity. The value of K_obs,Mg_ (the observed dissociation constant for two magnesium ions from the Exo I complex), was used to compare metal ion binding affinities of MszExo I and *E. coli* Exo I. Activities of the two enzymes were measured in the presence of various concentrations of MgCl_2_ by FRET-based exonuclease activity assay [18]. Compared with the traditional gel electrophoresis-based assay using radiolabelled substrates, this FRET-based assay can provide kinetic parameters conveniently. The initial velocities were plotted as a function of the concentration of MgCl_2_. The data fitted well to the binding equation for a single binding site (Fig. 4). The value of K_obs,Mg_ for MszExo I is 0.66 mM, comparable to that for *E. coli* Exo I (K_obs,Mg_ = 0.38 mM). It indicates these two enzymes have similar metal ion binding affinities.

### Broader working temperature and higher thermal stability of MszExo I

**Fig 5:**
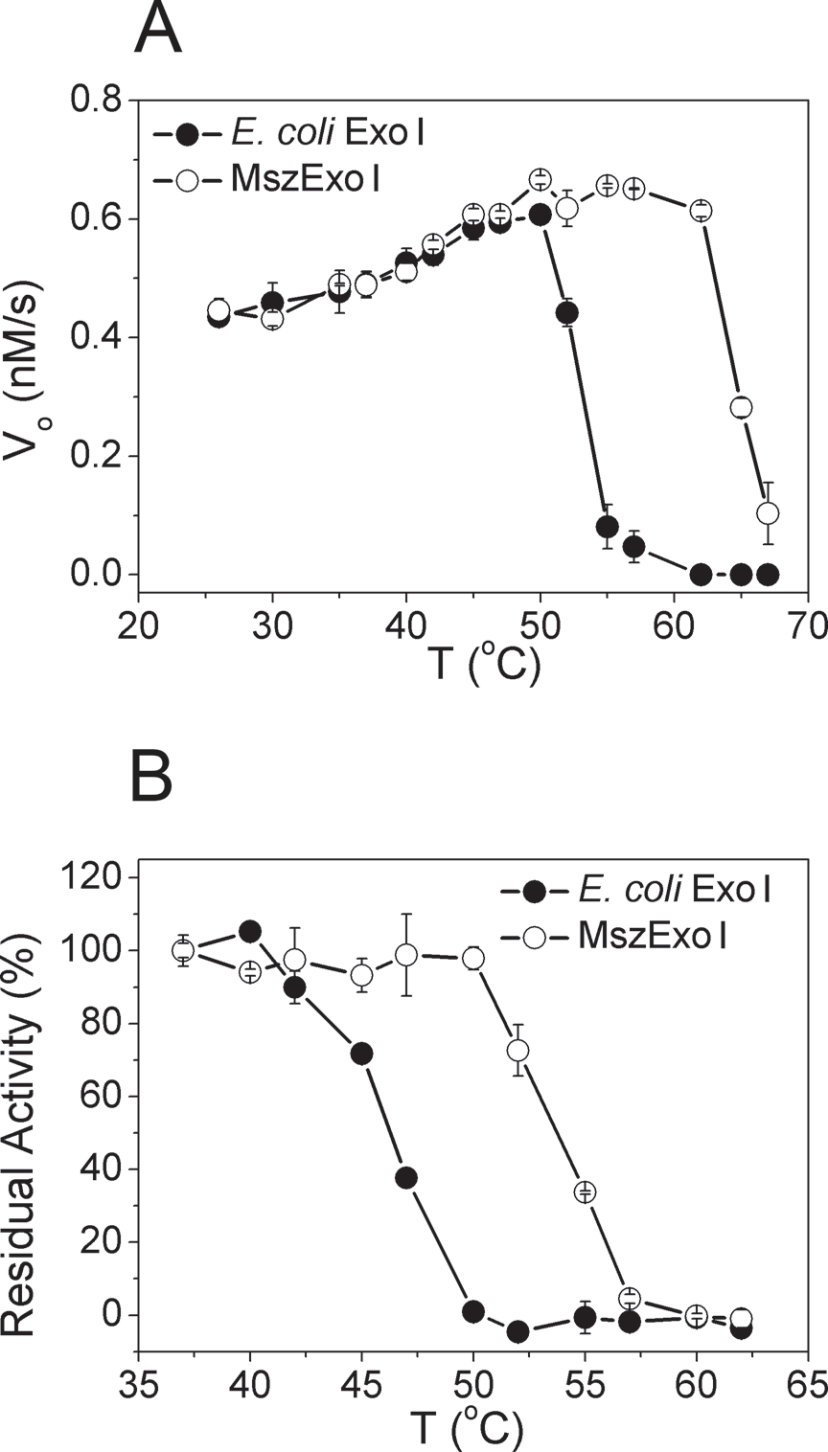
Thermal profiling of *E. coli* Exo I and MszExo I. (A) Determining optimum working temperature for two Exo Is. The reaction conditions were 5 nM enzyme, 1 μM ssDNA substrate (half FRET-dT67 with half dT67), 50 mM Glycine-NaOH buffer, pH 9.5, 200 mM NaCl, 0.1 mg/ml BSA and 5mM MgCl_2_ at given temperature varying from 25 to 67°C. Other details are described in Materials and Methods. MszExo I (open circle) showed broader working temperature than *E. coli* Exo I (filled circle). Errors were calculated from three parallel experiments. (B) Thermal stability of two Exo Is evaluated by thermal inactivation assay. In this assay, 15 nM enzyme in standard reaction buffer was incubated at given temperature varying from 25 to 62°C for 10 min, then cooled on ice, and finally activity was determined at 37°C. The data were normalized by taking the activity of enzymes incubated at 37°C as 100%. Errors came from three parallel experiments. Thermal stability of MszExo I was by 5°C higher than that of *E. coli* Exo I.

MszExo I is predicted to have better thermal stability than its *E. coli* counterpart since it was cloned from a moderately thermophilic strain. To test this idea, exonuclease activities were measured over a temperature range of 25–67°C (Fig. 5A) to derive the optimum working temperature for the respective exonucleases. The protein concentration was 5 nM. Up to 47°C, MszExo I showed similar activity to *E. coli* Exo I. The activity of *E. coli* Exo I dropped rapidly at temperatures over 50°C, and was completely inactivated at around 60°C. However, during this range (50–60°C), MszExo I maintained high exonuclease activity, with activity decreasing only at temperatures above 60°C.

To further characterize MszExo I, thermal inactivation assays were performed. Though the half-life of *E. coli* Exo I at 37°C was longer than six hours (data not shown), its activity began to decrease after incubating at 42°C for 10 minutes (Fig. 5B). It was completely inactivated after being incubated at 50°C for 10 min. In the case of MszExo I, higher thermal stability was observed. MszExo I retained most of its activity even when incubated at 50°C for 10 min, and was completely inactivated at nearly 60°C.

**Fig 6:**
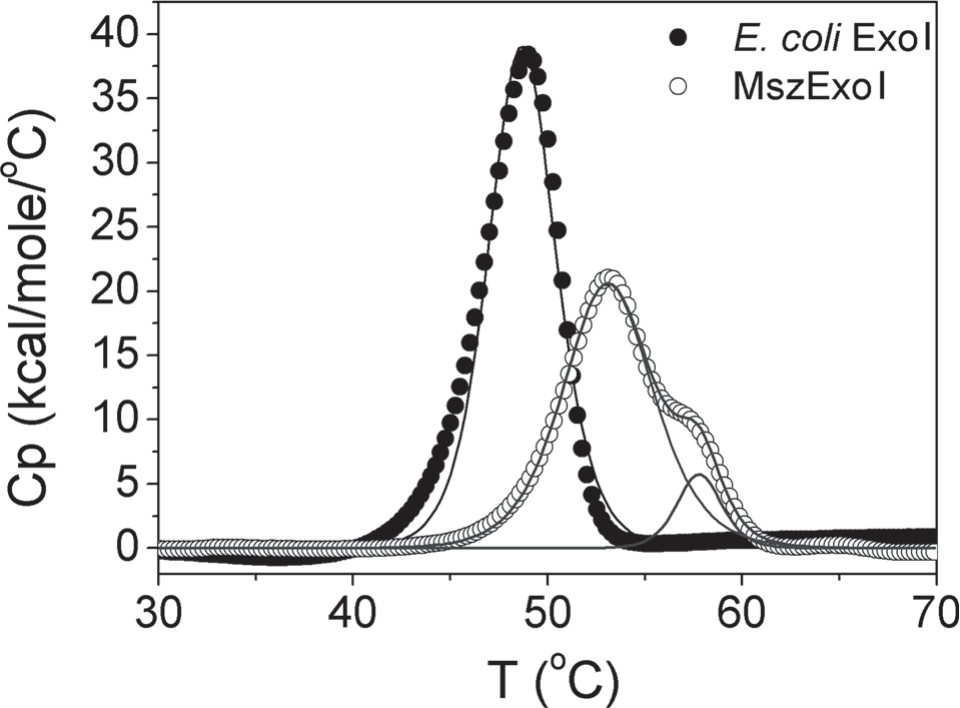
Thermal stability of *E. coli* Exo I and MszExo I evaluated by DSC. Comparison of DSC curve of *E. coli* Exo I (filled circle) and MszExo I (open circle). The protein concentration was 70 μM. The *E. coli* Exo I data (filled circle) were fitted to two-state transition model (black solid line). The T_m_ value for *E. coli* Exo I was 49°C. The MszExo I data (open circle) were fitted to non-two-state transition model (grey solid line). Two peaks indicated the two individual transitions. T_m_ values of these two transitions were 53.1°C and 57.4°C, respectively.

Thermal stabilities of the two enzymes were further compared by DSC measurements. *E. coli* Exo I exhibited a cooperative two-state transition at 49.0°C (T_m_) while MszExo I followed a non-two-state transition with calculated T_m_ values at 53.1°C and 57.4°C (Fig. 6). Higher T_m_ value of MszExo I indicates a higher thermal stability, consistent with the result from thermal inactivation assays. MszExo I has two unfolding entities melting at different temperatures, as indicated by the fitted data while *E. coli* Exo I only has one (Fig. 6). It is interesting to observe that the two enzymes displayed different unfolding profile, especially considering the sequence similarity and 3D structure similarities.

### Magnesium ion modulates thermal unfolding curve of MszExo I

**Fig 7:**
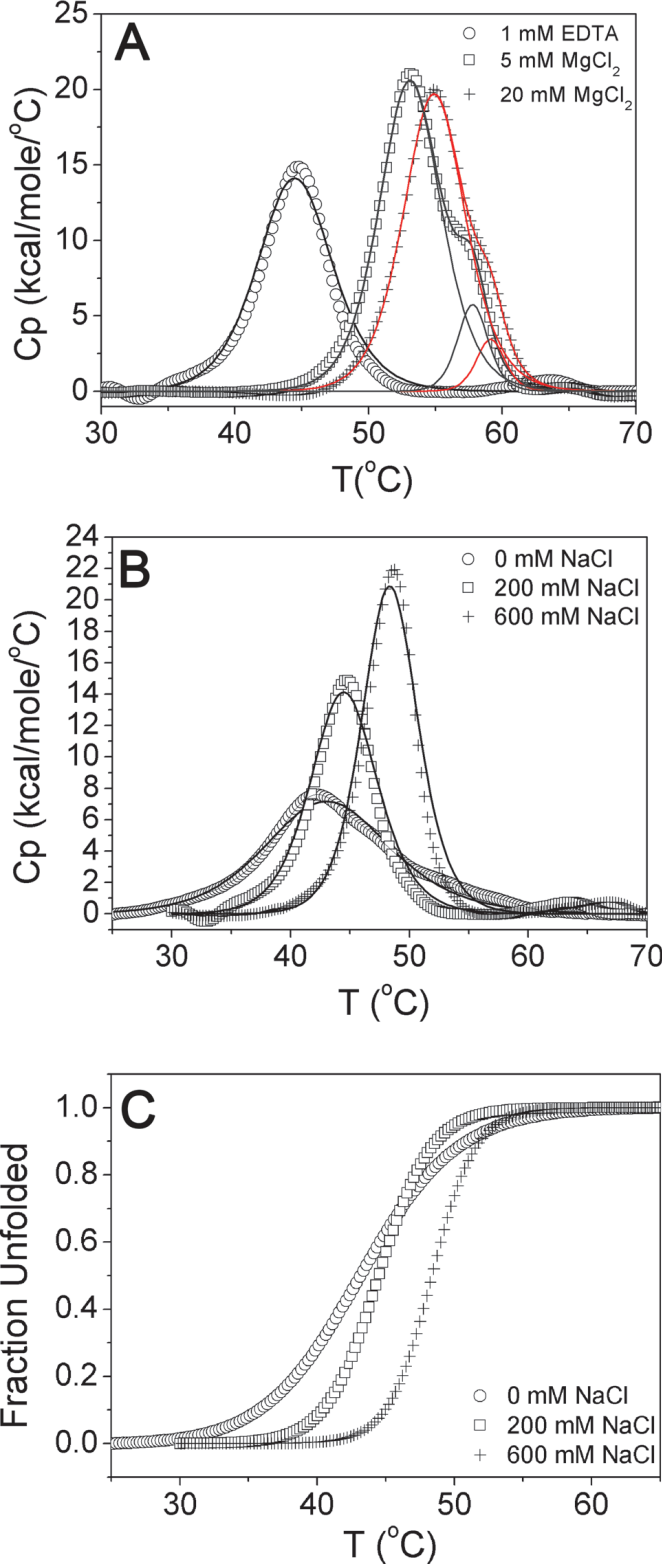
DSC measurements of MszExo I under different conditions. (A) DSC measurements of MszExo I as a function of magnesium ions concentration. DSC measurements of MszExo I in three different buffers were performed. The base buffer was 50 mM Glycine-NaOH, 200 mM NaCl, pH 9.5. Open circle represents the base buffer in the presence of 1mM EDTA, and black line represents the best fit to two-state transition model. Open square represents the base buffer in addition of 5 mM MgCl_2_, and grey line represents the best fit to non-two-state transition model. Plus sign represents the base buffer in addition of 20 mM MgCl_2_, and red line represents the best fit to non-two-state transition model. All Tm values are shown in Table 2. (B) DSC measurements of MszExo I as a function of salt concentration. DSC measurements of MszExo I in three different buffers were performed. The base buffer was 50 mM Glycine-NaOH, 1 mM EDTA, pH 9.5. Open circle represents the base buffer, open square represents the base buffer in addition of 200 mM NaCl, and plus sign represents the base buffer in addition of 600 mM NaCl. DSC data were fitted to two-state transition model. Tm values are shown in Table 2. (C) The unfolded fraction of MszExo I monitored by the transition enthalpy. DSC Data in Fig. 7B were transformed into F_U_-T plots by integration of the Cp versus T curve.

To further investigate the unfolding transitions of MszExo I, DSC measurements were performed as a function of concentration of magnesium ions. In the presence of 1 mM EDTA, single thermal transition was observed (Fig. 7A). In the presence of 5 mM MgCl_2_, the T_m_ value increased by more than 8°C, and what is more, two peaks representing two unfolding transitions were clearly observed. If the concentration of MgCl_2_ was further increased to 20 mM, the second peak became smaller and appeared as a shoulder. According to the fitted data, two unfolding entities were also clearly observed under this condition (Fig. 7A). Meanwhile, the T_m_ value of the first transition increased by 1.7 degrees (Table 2). These results indicate the magnesium ion plays multiple roles during thermal unfolding of MszExo I. It could not only stabilize the whole structure of MszExo I, but also affect unfolding entities or unfolding intermediates as well.

**Table 2:** Tm value of MszExo I in different buffers.

Buffer	T_m1_ (°C)	T_m2_ (°C)
1 mM EDTA + B1*	44.6	No
5 mM MgCl_2_ + B1	53.1	57.4
20 mM MgCl_2_ + B1	54.8	58.4
0 mM NaCl + B2**	43.1	No
200 mM NaCl + B2	44.6	No
600 mM NaCl + B2	48.5	No

*, B1 is the buffer which is composed of 50 mM Glycine-NaOH, 200 mM NaCl, pH 9.5.

**, B2 is the buffer which is composed of 50 mM Glycine-NaOH, 1 mM EDTA, pH 9.5.

To rule out the possibility that the appearance of two unfolding transitions of MszExo I was caused by increased ionic strength of buffers with 5 mM MgCl_2_ or 20 mM MgCl_2_, DSC measurements were performed as a function of salt concentration (Fig. 7B). As the figure shows, MszExo I always exhibited single peak during thermal unfolding in buffers with different concentration of salts, though higher T_m_ value with increased salt concentration was also observed (Fig. 7B & Table 2). Besides, the unfolding transition of MszExo I in the presence of 200 mM NaCl and 600 mM NaCl was highly cooperative. However, in the absence of sodium chloride, the unfolding transition became less cooperative (Fig. 7C), indicating salts are essential for the stability of MszExo I. MszExo I began to unfold after 35°C without sodium chloride, which is consistent with the fact that MszExo I showed poor activity and thermal stability in the Exo I commercial buffer which contains barely no salt.

Taken together, the role of magnesium ions in thermal unfolding of MszExo I is unique and could not be replaced by salts.

## Discussion

Exo I was first characterized in *E. coli* by Lehman and Nussbaum [1]. It is conserved in bacteria, but not in archaea and eukarya. Recently, a hyperthermophilic exonuclease I, PfuExo I, was identified, though with limited sequence similarity to *E. coli* Exo I [14]. It digests ssDNA at every two nucleotides, different from the catalytic mechanism of *E. coli* Exo I. Considering this fact, moderately thermophilic bacteria seemed a productive source for finding robust alternatives to *E. coli* Exo I.

In bacteria, the nucleic acids metabolic pathways of *E. coli* and *Bacillus subtilis* have been well studied [19,20], and those of archaea are emerging [21–23]. However, few reports on the DNA metabolic pathways of moderately thermophilic bacteria have been published, probably due to the small population of moderately thermophilic bacteria being found. However, more and more moderately thermophilic bacteria have been described recently, in many cases as potential sources of useful enzymatic activities [24–28]. For example, the keratinolytic protease from *Meiothermus ruber* H328 shows extraordinary tolerance to denaturants [28]. Several genomes of moderately thermophilic bacteria have been completely sequenced [29,30]. Therefore, more studies of moderately thermophilic bacteria are expected.

Our report is the first characterizing an enzyme involved in a DNA repair system from a moderately thermophilic bacteria. At higher temperatures the amount of DNA damage will increase, hence repair systems are required to be more efficient to ensure the faithful transmission of genomic DNA to the next generation. More research is needed to elucidate the differences in DNA repair systems from moderately thermophilic bacteria and mesophilic ones, as well as the similarities. In our study, we found that MszExo I exhibits similar metal ion binding affinity and specific activity as *E. coli* Exo I. However, during thermal denaturation different unfolding pathways were observed even though their crystal structures are highly conserved. This may be related to the high performance of MszExo I at moderately thermophilic temperatures. Obviously, *in vivo* studies on the role of MszExo I in DNA repair systems of *Methylocaldum szegediense* would be informative to demonstrate whether MszExo I functions similarly to its *E. coli* counterpart or has extra functions.

## Supporting Information

S1 File Preparation of MszExo I protein for crystallization. (DOCX)

S1 Fig Sequence alignment of E. coli Exo I and MszExo I. Identical residues are labeled by ‘*’, and similar residues are labeled by ‘:’ or ‘.’. Residues at the active site are highlighted in yellow, and those at the anchor site are highlighted in red. Sequence alignment was performed by CLUSTALW program.
(TIF)

S2 Fig Pufication of MszExo I. The purified MszExo I protein (4 μg) was subjected to 10% SDS-PAGE followed by Commassie Brilliant Blue staining. The yield of MszExo I was around 1–2 mg per liter culture. Marker, color protein standard (broad range) (New England Biolabs).
(TIF)

## References

[1] Lehman IR, Nussbaum AL (1964) The Deoxyribonucleases of Escherichia Coli. V. On the Specificity of Exonuclease I (Phosphodiesterase). J Biol Chem 239: 2628–2636. 14235546

[2] Brody RS, Doherty KG, Zimmerman PD (1986) Processivity and kinetics of the reaction of exonuclease I from Escherichia coli with polydeoxyribonucleotides. J Biol Chem 261: 7136–7143. 3519606

[3] Burdett V, Baitinger C, Viswanathan M, Lovett ST, Modrich P (2001) In vivo requirement for RecJ, ExoVII, Exo I, and ExoX in methyl-directed mismatch repair. Proc Natl Acad Sci U S A 98: 6765–6770. 11381137 10.1073/pnas.121183298 PMC34427

[4] Feschenko VV, Rajman LA, Lovett ST (2003) Stabilization of perfect and imperfect tandem repeats by single-strand DNA exonucleases. Proc Natl Acad Sci U S A 100: 1134–1139. 12538867 10.1073/pnas.0233122100 PMC298739

[5] Breyer WA, Matthews BW (2000) Structure of Escherichia coli exonuclease I suggests how processivity is achieved. Nat Struct Biol 7: 1125–1128. 11101894 10.1038/81978

[6] Korada SK, Johns TD, Smith CE, Jones ND, McCabe KA, et al (2013) Crystal structures of Escherichia coli exonuclease I in complex with single-stranded DNA provide insights into the mechanism of processive digestion. Nucleic Acids Res 41: 5887–5897. 10.1093/nar/gkt278 23609540 PMC3675492

[7] Busam RD (2008) Structure of Escherichia coli exonuclease I in complex with thymidine 5'-monophosphate. Acta Crystallogr D Biol Crystallogr 64: 206–210. 10.1107/S090744490706012X 18219121

[8] Steitz TA, Steitz JA (1993) A general two-metal-ion mechanism for catalytic RNA. Proc Natl Acad Sci U S A 90: 6498–6502. 8341661 10.1073/pnas.90.14.6498 PMC46959

[9] Beese LS, Steitz TA (1991) Structural basis for the 3'-5' exonuclease activity of Escherichia coli DNA polymerase I: a two metal ion mechanism. EMBO J 10: 25–33. 1989886 10.1002/j.1460-2075.1991.tb07917.x PMC452607

[10] Yang W, Lee JY, Nowotny M (2006) Making and breaking nucleic acids: two-Mg2+-ion catalysis and substrate specificity. Mol Cell 22: 5–13. 16600865 10.1016/j.molcel.2006.03.013

[11] Liu WF, Zhang A, Cheng Y, Zhou HM, Yan YB (2007) Effect of magnesium ions on the thermal stability of human poly(A)-specific ribonuclease. FEBS Lett 581: 1047–1052. 17306797 10.1016/j.febslet.2007.02.008

[12] Bernad A, Blanco L, Lazaro JM, Martin G, Salas M (1989) A conserved 3'——5' exonuclease active site in prokaryotic and eukaryotic DNA polymerases. Cell 59: 219–228. 2790959 10.1016/0092-8674(89)90883-0

[13] Clarke J, Wu HC, Jayasinghe L, Patel A, Reid S, et al (2009) Continuous base identification for single-molecule nanopore DNA sequencing. Nat Nanotechnol 4: 265–270. 10.1038/nnano.2009.12 19350039

[14] Tori K, Ishino S, Kiyonari S, Tahara S, Ishino Y (2013) A novel single-strand specific 3'-5' exonuclease found in the hyperthermophilic archaeon, Pyrococcus furiosus. PLoS One 8: e58497 10.1371/journal.pone.0058497 23505520 PMC3591345

[15] Bodrossy L, Holmes EM, Holmes AJ, Kovacs KL, Murrell JC (1997) Analysis of 16S rRNA and methane monooxygenase gene sequences reveals a novel group of thermotolerant and thermophilic methanotrophs, Methylocaldum gen. nov. Arch Microbiol 168: 493–503. 9385141 10.1007/s002030050527

[16] Didenko VV (2001) DNA probes using fluorescence resonance energy transfer (FRET): designs and applications. Biotechniques 31: 1106–1116, 1118, 1120–1101. 11730017 10.2144/01315rv02 PMC1941713

[17] Takacs E, Grolmusz VK, Vertessy BG (2004) A tradeoff between protein stability and conformational mobility in homotrimeric dUTPases. FEBS Lett 566: 48–54. 15147867 10.1016/j.febslet.2004.04.039

[18] Ghosh SS, Eis PS, Blumeyer K, Fearon K, Millar DP (1994) Real time kinetics of restriction endonuclease cleavage monitored by fluorescence resonance energy transfer. Nucleic Acids Res 22: 3155–3159. 8065930 10.1093/nar/22.15.3155 PMC310290

[19] Wigley DB (2013) Bacterial DNA repair: recent insights into the mechanism of RecBCD, AddAB and AdnAB. Nat Rev Microbiol 11: 9–13. 10.1038/nrmicro2917 23202527

[20] Lenhart JS, Schroeder JW, Walsh BW, Simmons LA (2012) DNA repair and genome maintenance in Bacillus subtilis. Microbiol Mol Biol Rev 76: 530–564. 10.1128/MMBR.05020-11 22933559 PMC3429619

[21] van Wolferen M, Ajon M, Driessen AJ, Albers SV (2013) How hyperthermophiles adapt to change their lives: DNA exchange in extreme conditions. Extremophiles 17: 545–563. 10.1007/s00792-013-0552-6 23712907

[22] Li Z, Kelman LM, Kelman Z (2013) Thermococcus kodakarensis DNA replication. Biochem Soc Trans 41: 332–338. 10.1042/BST20120303 23356307

[23] Frols S, White MF, Schleper C (2009) Reactions to UV damage in the model archaeon Sulfolobus solfataricus. Biochem Soc Trans 37: 36–41. 10.1042/BST0370036 19143598

[24] Slobodkina GB, Kolganova TV, Tourova TP, Kostrikina NA, Jeanthon C, et al (2008) Clostridium tepidiprofundi sp. nov., a moderately thermophilic bacterium from a deep-sea hydrothermal vent. Int J Syst Evol Microbiol 58: 852–855. 10.1099/ijs.0.65485-0 18398181

[25] Iino T, Nakagawa T, Mori K, Harayama S, Suzuki K (2008) Calditerrivibrio nitroreducens gen. nov., sp. nov., a thermophilic, nitrate-reducing bacterium isolated from a terrestrial hot spring in Japan. Int J Syst Evol Microbiol 58: 1675–1679. 10.1099/ijs.0.65714-0 18599715

[26] Ueda J, Yamamoto S, Kurosawa N (2013) Paenibacillus thermoaerophilus sp. nov., a moderately thermophilic bacterium isolated from compost. Int J Syst Evol Microbiol 63: 3330–3335. 10.1099/ijs.0.048090-0 23504971

[27] You ZQ, Li J, Qin S, Tian XP, Wang FZ, et al (2013) Georgenia sediminis sp. nov., a moderately thermophilic actinobacterium isolated from sediment. Int J Syst Evol Microbiol 63: 4243–4247. 10.1099/ijs.0.051714-0 23811137

[28] Kataoka M, Yamaoka A, Kawasaki K, Shigeri Y, Watanabe K (2013) Extraordinary denaturant tolerance of keratinolytic protease complex assemblies produced by Meiothermus ruber H328. Appl Microbiol Biotechnol. 10.1007/s00253-013-5155-8 23955472

[29] Anderson I, Chertkov O, Chen A, Saunders E, Lapidus A, et al (2012) Complete genome sequence of the moderately thermophilic mineral-sulfide-oxidizing firmicute Sulfobacillus acidophilus type strain (NAL(T)). Stand Genomic Sci 6: 1–13. 10.4056/sigs.2736042 23407703 PMC3558970

[30] Goker M, Saunders E, Lapidus A, Nolan M, Lucas S, et al (2012) Genome sequence of the moderately thermophilic, amino-acid-degrading and sulfur-reducing bacterium Thermovirga lienii type strain (Cas60314(T)). Stand Genomic Sci 6: 230–239. 10.4056/sigs.2726028 22768366 PMC3387794

