# Supplementary material for: Structural and Biochemical Studies of a Moderately Thermophilic Exonuclease I from Methylocaldum szegediense
Source: PLoS One. 2015 Feb 6;10(2):e0117470. doi: 10.1371/journal.pone.0117470 (PMC4319927; doi:10.1371/journal.pone.0117470)
Supplement: S1 File — (DOCX) [file pone.0117470.s001.docx]

For crystallization studies the MszExo I gene was synthesized with an N-terminal cleavable strep tag. The synthetic gene was cloned into the Pt7 expression vector and transformed into BL21 (DE3). Cultures were grown at 37 ^o^C in terrific broth (<http://cshprotocols.cshlp.org/content/2006/1/pdb.rec8620>) containing 100 μg/ml ampicillin until OD600 value reached 0.6. Expression of recombinant protein was induced by addition of 1 mM IPTG at 18 ^o^C for 16 hours. Cells were harvested and lysed using bugbuster (Novagen). The cell lysate was clarified by centrifugation and protein purified using a StrepTrap column (GE Healthcare Life Sciences) eluting with desthiobiotin. The strep tag was then removed by cleavage with a strep tagged TEV protease and any uncleaved material and TEV removed by passing the sample through a StrepTrap column. The material was further purified by loading on a heparin column (GE Healthcare Life Sciences) and eluting with a linear gradient of 0-1M NaCl. The sample was desalted prior to crystallization.
