# Supplementary figures and images for: Structural and Biochemical Studies of a Moderately Thermophilic Exonuclease I from Methylocaldum szegediense
Source: PLoS One. 2015 Feb 6;10(2):e0117470. doi: 10.1371/journal.pone.0117470 (PMC4319927; doi:10.1371/journal.pone.0117470)

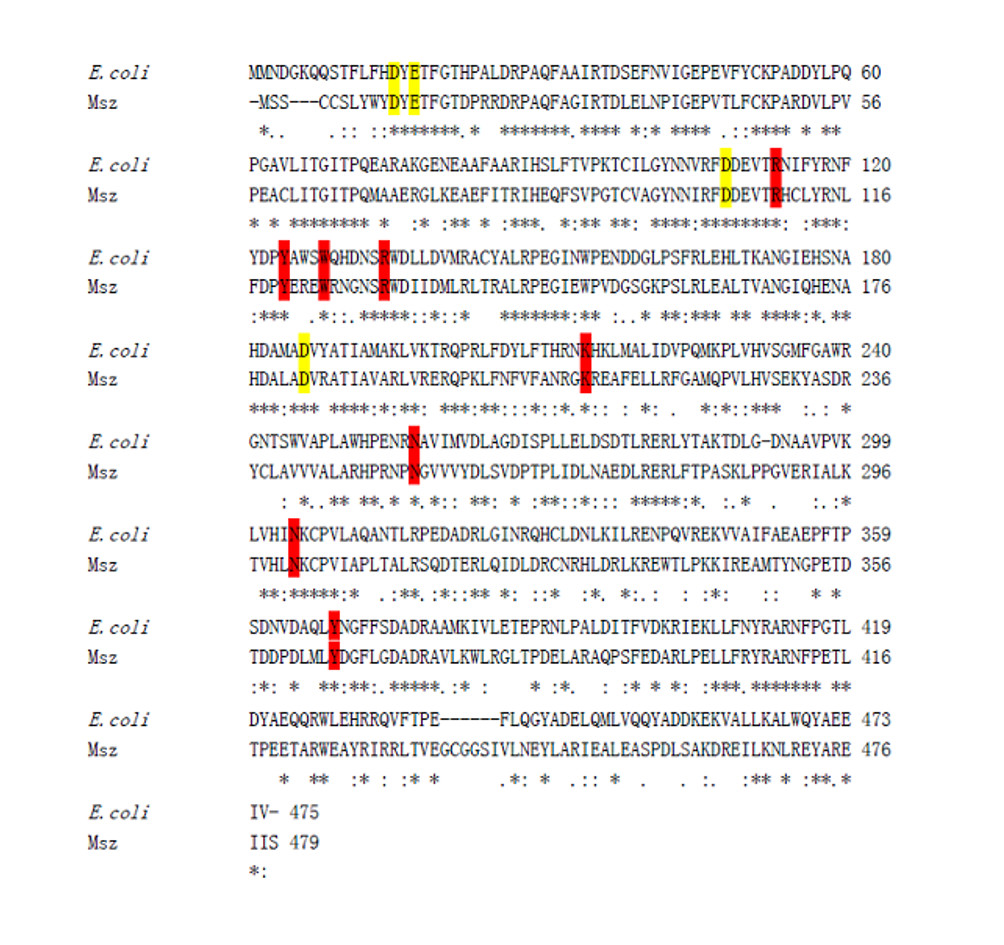

Supplement: S1 Fig — Identical residues are labeled by ‘*’, and similar residues are labeled by ‘:’ or ‘.’. Residues at the active site are highlighted in yellow, and those at the anchor site are highlighted in red. Sequence alignment was performed by CLUSTALW program. (TIF) [file pone.0117470.s002.tif]

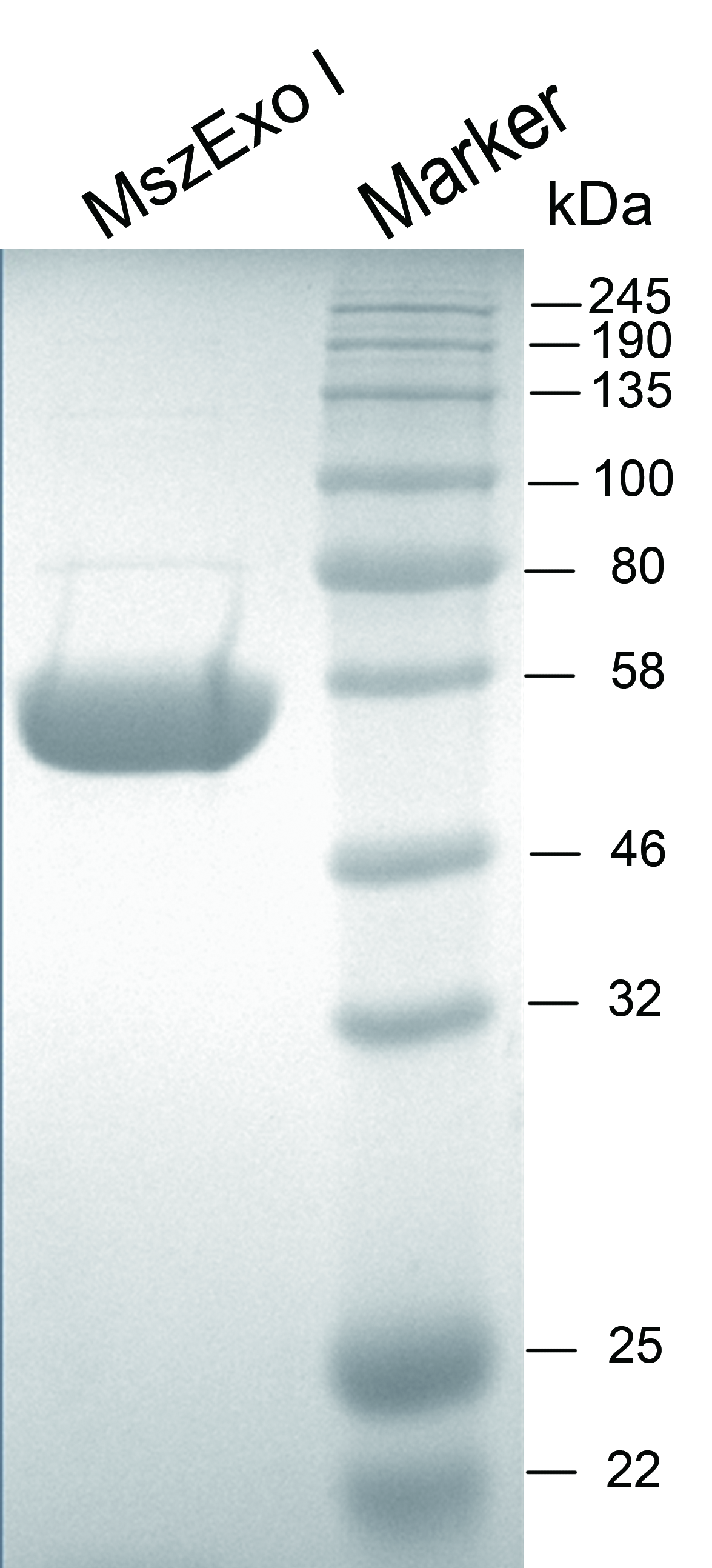

Supplement: S2 Fig — The purified MszExo I protein (4 μg) was subjected to 10% SDS-PAGE followed by Commassie Brilliant Blue staining. The yield of MszExo I was around 1–2 mg per liter culture. Marker, color protein standard (broad range) (New England Biolabs). (TIF) [file pone.0117470.s003.tif]
